# Response to “Comment on ‘Rheumatoid Arthritis in Agricultural Health Study Spouses: Associations with Pesticides and Other Farm Exposures’”

**DOI:** 10.1289/EHP944

**Published:** 2016-11-01

**Authors:** Christine G. Parks, Jane A. Hoppin, Anneclaire J. De Roos, Karen H. Costenbader, Dale P. Sandler

**Affiliations:** 1 Epidemiology Branch, National Institute of Environmental Health Sciences, National Institutes of Health, Department of Health and Human Services, Research Triangle Park, North Carolina, USA; 2 Center for Human Health and the Environment, Department of Biological Sciences, North Carolina State University, Raleigh, North Carolina, USA; 3 Department of Environmental and Occupational Health, Drexel University School of Public Health, Philadelphia, Pennsylvania, USA; 4 Brigham and Women’s Hospital, Harvard Medical School, Boston, Massachusetts, USA.

We appreciate the comments about our recent paper on pesticides and other farming exposures in relation to rheumatoid arthritis (RA) in female spouses in the Agricultural Health Study (AHS). In their letter, Murphy et al. suggest that heavy metals, such as manganese and cadmium, may account for observed associations of RA with the fungicide maneb/mancozeb and chemical fertilizers, and note other relevant sources of manganese and cadmium exposure. It is intriguing to consider the potential role of metals in RA. As the authors noted, maneb/mancozeb are 21% manganese by weight, and it is possible that this is the relevant active ingredient responsible for the observed association. It is important to note, however, that it is unlikely that metals exposure accounts for the other observed pesticide associations in our study, including those with glyphosate and DDT. In our study sample, maneb/mancozeb use was very uncommon (less than 1% of the cohort and 4% of RA cases) and was not correlated with the other pesticides (e.g., glyphosate, DDT) associated with RA.

As Murphy et al. point out, chemical fertilizers may contain cadmium as a contaminant from phosphate mining. This is an important consideration, although levels of cadmium and other metals in commercial products are highly variable (Jiao et al. 2012), so individual exposure levels may be difficult to determine based on self-reported use alone. Other than dietary exposure from foods grown in contaminated soils, there is little known about farmers’ exposure to metals from soil dust. In addition to chemical fertilizers, organic manures may also be a source of heavy metals contributing to increased levels in treated soils (Zhou et al. 2015). In the United States, work in agriculture has been associated with lower urinary cadmium levels compared with several other industries, including repair service, metals manufacturing, construction, and transportation (Yassin and Martonik 2004). Murphy et al. also note that other sources of occupational and nonoccupational metals exposures were associated with RA in our paper.

Accurately characterizing metals exposures in the AHS will require considering potential exposures from on- and off-farm jobs, pesticides, fertilizers, and natural sources, e.g., in the soil. We see parallels in assessing another established RA risk factor, crystalline silica (Miller et al. 2012), which occurs naturally in soils, with exposures and silica-related pathology similar to those seen in the “dusty trades” industries (Archer et al. 2002; Schenker et al. 2009). Data needed to assess agricultural silica exposure include tasks performed, crops grown, and soil characteristics (Parks et al. 2003). In the AHS, we expect participants to have a range of exposures to metals such as cadmium, manganese, lead, iron, and arsenic, as well as to silica. Questionnaire data can be used to estimate metals exposures from occupational sources and farm tasks, crops and geocoded farm residence data linked to soil mapping data. Challenges include limited spatial resolution in soil measurements and unmeasurable variation due to fertilizers and other soil amendments (Nicholson et al. 2003). Past practices such as the use of lead arsenate pesticides also may contribute to localized higher concentrations, for example in orchards (Wolz et al. 2003). Soil concentrations of metals and minerals are often correlated because they reflect the composition of the parent rock, highlighting a need to consider multiple exposures (Davis et al. 2009). Future AHS analyses will explore the contribution of occupational factors and soil characteristics to the development of RA, in addition to continued research on the role of pesticides and other farming-related exposures.
